# Supplementary material for: Gene-by-environment interactions influence the fitness cost of gene copy-number variation in yeast
Source: G3 (Bethesda). 2023 Jul 22;13(10):jkad159. doi: 10.1093/g3journal/jkad159 (PMC10542507; doi:10.1093/g3journal/jkad159)
Supplement: jkad159_Supplementary_Data [file jkad159_supplementary_data.zip › Supplemental_Material_Legends_G3-2023-404394.docx]

**Robinson et al. G3. 2023**

**DATASET LEGENDS

Dataset S1: Normalized and imputed barcode read count.** Normalized and imputed data from MoBY 2.0 selection experiments, as described in Methods.

**Dataset S2: *EdgeR* output for MoBY 2.0 selections.** Each block represents edge R results for each indicated strain, see *edgeR* manual for details.

**Dataset S3: Beneficial genes.** Each tab represents *edgeR* output for genes determined to be significant at FDR < 0.05. Tabs for BY4743 and BC187 labeled ‘threshold’ are those with FDR < 0.05 and meeting the effect size threshold as described in Methods.

**Dataset S4: Normalized log2 relative gene expression from RNA-seq experiments.** Tab 1) log_2_ values represent the fold change in expression in each denoted strain at 30 min or 3 h versus the unstressed sample for that strain. Tab 2) log_2_ values represent the fold difference in expression in each denoted unstress strain relative to the mean expression of all four strains from that paired replicate.
